# Supplementary material for: Genome-Wide Identification, Characterization, and Expression Analysis of DDE_Tnp_4 Family Genes in Eriocheir sinensis
Source: Antibiotics (Basel). 2021 Nov 23;10(12):1430. doi: 10.3390/antibiotics10121430 (PMC8697882; doi:10.3390/antibiotics10121430)
Supplement: Supplementary file 1 [file antibiotics-10-01430-s001.zip › Supplementary Material.pdf]

Supplementary Materials

Genome-Wide Identification, Characterization, and Expression Analysis of DDE_Tnp_4 Family Genes in Eriocheir sinensis

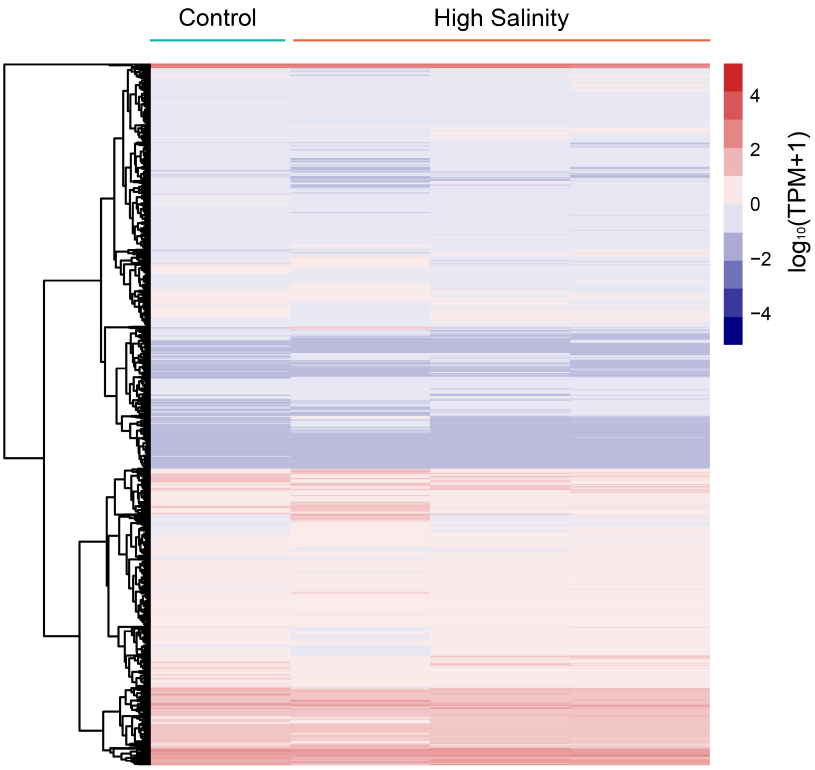

Figure S1. Global expression profiles of genes in *E. sinensis* hemocytes under acute high salinity conditions.

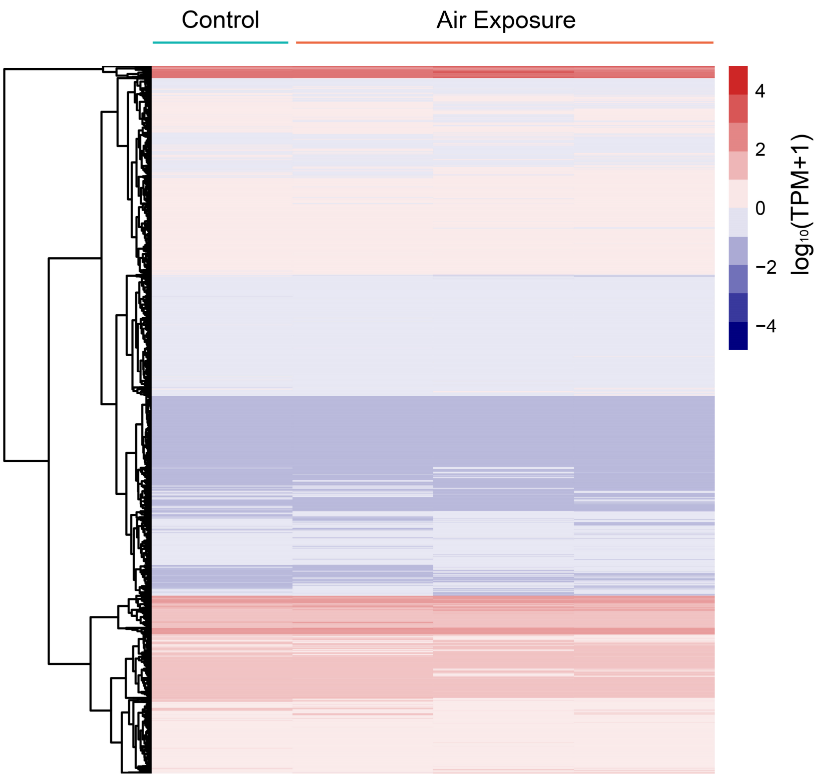

Figure S2. Global expression profiles of genes in *E. sinensis* gills under acute air exposure stress conditions.

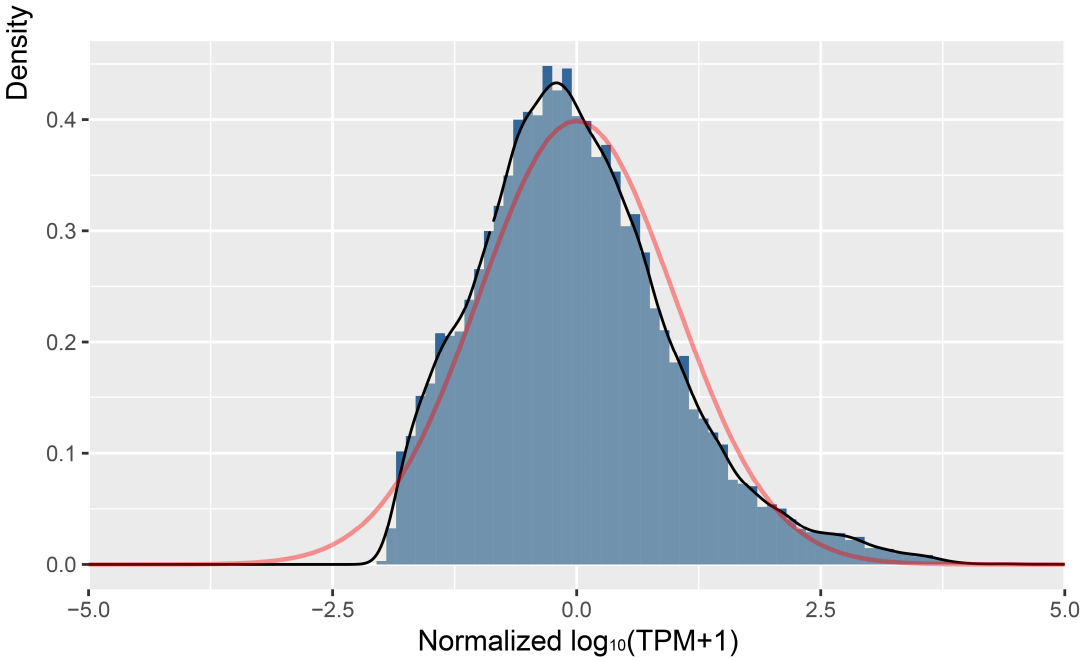

Figure S3. The normalization and distribution of gene expression in *E. sinensis* hemocytes under acute high salinity conditions.

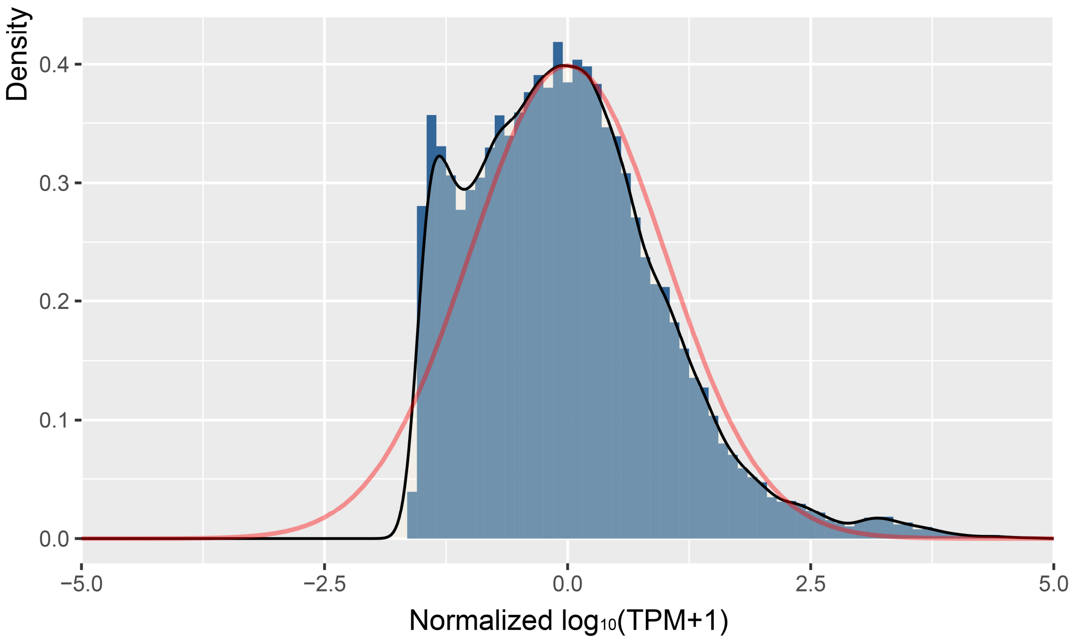

Figure S4. The normalization and distribution of gene expression in *E. sinensis* gills under air exposure stress conditions.

Sequence S1. Amino acid sequences of DDE_Tnp_4 family in *E. sinensis*, *S. paramamosain*, *P. trituberculatus*, and *D. pulex*.

>CCG015995.1
MEGREKITRERRGKWLANLARADLTEAGKRHLLVCSDHFLSGRPAVLEDTSNPDWALTQ
NLGHRKVSQELVAIICARHQRIQERKLKKDKEMFMAAQTLMDIEEYTQPCSDEVHVEQQE
QLSLPAIGQTFQTEKQTILSRPAACQTETVTVATVGCQTDLTLMEMTMFDQQYEDLNQLRT
EVYELRFTVGDYNNLNLSNDDEKVLFYTGLPNYAVLMCIFNYVCLNITHSINQSYPRFKNL
YVSDASSFKHMLPGFPTDLMYLFQLCQGYFYKWVDTADTKLRHSIVWPGREELRLAMPAC
FCRNFGDRVSVILDCFEMTLATPSSLMAKAATWSNYKHNNTMKFLIGITPTGCISFISRGWG
GRVSDKTITEKCGILENLLPGDIVLADRGFGIEESVGLYHASLYIPSFTRGKSQLSAFEVENTR
KIANVRIHVERVIGLVRRKYKIFQGPLPIELVKGNDSNETTLDKIVKDMLCTC
>CCG042713.1
MLLALCGTEPVSADVRGRPAVLEDTSPNPDWAPTQNLGHRKVSQELVAISCARHQRIQER
KLKKDKEMFMAAQTLMDIQEYTQPCSDEVHVEQQEQLSLPAIGQTFQTEKQTILSRPAAC
QTETVTVATVGCQTDLTLMEMTMFDQQYEDLNQLRTGVYELRSTVGDYNNLNLSNDDEK
VLFYTGLPNYAVLMCIFNYVCLNITHSINSKLSPFQEFVMFLMHLRLNTCFQDLAYIFNVSL
STVSRIFYKWVDTADTNLRHSIVWPGREELRLARNFGDRVSVILDCFEVTLATPSSLMAKAA
TWSNYKYNITMKFLIGITPTGCISFISRGWGGRVSDKTITEKCGILENLLPGDIVLADRGFGIE
ESVGLYHASLYIPSFTRGKSQLSAFEVENTRKIANVRIHVERVIGLVRRKYKI
>CCG026042.1
MPRHEILRLCDELSPLLSRATKRAHAIPTHTQVLVTLRFLASGTFQNVIADTTGLTQPSVSRII

SSVTNILYEKAKMEIKMPTNIYDVNRTAAAFSFIGGFPRVIGAIDCTHIPIKAPIENEHIYVNR
KSFHSLNVQVICNAEMLITSFSVKYPGATNDAFIWRNCPLRDRFEAGTFRDLHLLGDSGYP
LEPYLLTPFHDPMTEGERQYNRSHKITRVIIEQTFGVLKSRFRCLHRSGGSLQYDPKKCAKIA
ATCMWLHNRCIRRRIPMIAPVGNDDGMNNDNDIIHGDHNPTGQDVRREIVEGFFT
>CCG011549.1
MDQSSVSRAIDKVTQVLCVKASQEIKMPTTAIDINRAMQDFRRTGNFPRVIGAIDGTHIRIK
APEENEEIYVNRKQFHSLNIQAVGDTNNKIISYDTSYPGSTHDSFIWRHCALKQRFLAGHFG
DALLIGDSGYPLEPFLMTPVVHPTTPGEERYNQSHRRTRCIVERTFGILKSRFRCLHESGGSL
QYDPEKTMKIATSCMLLHNYCVDRRIPYVGDLVQEEEVPVQPVRDNRQVPGQVVRQEIIR
NFFS
>CCG030787.1
MEYVAVYVFNRRLRRERRYRDPLDPLHVSDEHLLRVYRFPRQEIIRLCDELRPHLERRTRRA
HALPTHTQVLAALRFFASGSFQTVIGDTVGMDQSSVSRAIDKVTQVLCVKASQEIKMPTTA
IDINRAMQDFRRTGNFPRVIGAIDGTHIRIKAPEENEEIYVNRKQFHSLNIQAVGDTNNKIIS
YDTSYPGSTHDSFIWRHCALKQRFLAGHFGDALLIGDSGYPLEPFLMTPVVHPTTPGEERY
NQSHRRTRCIVERTFGILKSRFRCLHESGGSLQYDPEKTMKIATSCMLLHNYCVDRRIPYVG
DLVQEEEVPVQPVRDNRQVPGQVVRQEIIRNFFS
>CCG035097.1
MMAALQVFEELDVLEDAAGERVRLPRRIVKDRLDLFLTLTEEEFTSRFRISKQSARALLADL
HLPEAADARGCPVPPHLQLLITLRWMATGDLHQTIGDCLDVSQQFVSSCSSHTVRAIAALY
QRYVKFPGRHELGSIKTNFEAISGFPGVIGAIDCTHIPILSPGGQQAETYRSRKGFFSLNVQA
VCGPDLTFYNIICRWPGSVHDSRIFANSSLYAQLQAGDYNGHLLGDSAYPLRPFLMTPVGN
PSRPNEGRYNLAHAKTRNVIERAFGVWKRRFRCLAIPMRTSLDTTMATICSAAVLHNIAM
ADRNEDMEDYDNDIINEEERLIENAQNRHAGQRKRANMINNVFSNE
>CCG046435.1
MMAALQVFEELDVLEDAAGERVRLPRRIVKDRLDLFLTLTEEEFTSRFRISKQSARALLADL
HLPEAADARGCPVPPHLQLLITLRWMATGDLHQTIGDCLDVSQQFVSSCSSHTVRAIAALY
QRYVKFPGRHELGSIKTNFEAISGFPGVIGAIDCTHIPILSPGGQQAETYRSRKGFFSLNVQA
VCGPDLTFYNIICRWPGSVHDSRIFANSSLYAQLQAGDYNGHLLGDSAYPLRPFLMTPVGN
PSRPNEGRYNLAHAKTRNVIERAFGVWKRRFRCLAIPMRTSLDTTMATICSAAVLHNIAM
ADRNEDMEDYDNDIINEEERLIENAQNRHAGQRKRANMINNVFSNE
>CCG073988.1
MMAALQVFEELDVLEDAAGERVRLPRRIVKDRLDLFLTLTEEEFTSRFRISKQSARALLADL
HLPEAADARGCPVPPHLQLLITLRWMATGDLHQTIGDCLDVSQQFVSSCSSHTVRAIAALY
QRYVKFPGRHELGSIKTNFEAISGFPGVIGAIDCTHIPILSPGGQQAETYRSRKGFFSLNVQA
VCGPDLTFYNIICRWPGSVHDSRIFANSSLYAQLQAGDYNGHLLGDSAYPLRPFLMTPVGN
PSRPNEGRYNLAHAKTRNVIERAFGVWKRRFRCLAIPMRTSLDTTMATICSAAVLHNIAM
ADRNEDMEDYDNDIINEEERLIENAQNRHAGQRKRANMINNVFSNE
>CCG011535.1
MMAALQVFEELDVLEDAAGERVRLPRRIVKDRLDLFLTLTEEEFTSRFRISKQSARALLADL
HLPEAADARGCPVPPHLQLLITLRWMATGDLHQTIGDCLDVSQQFVSSCSSRTVRAIAALY
QRYVKFPGRHELGSIKTNFEAISGFPGVIGAIDCTHIPILSPGGQQAETYRSRKGFFSLNVQA
VCGPDLTFYNIICRWPGSVHDSRIFANSSLYAQLQAGDYNGHLLGDSAYPLRPFLMTPVGN
PSRPNEGRYNLAHAKTRNVIERAFGVWKRRFRCLAIPMRTSLDTTMATICSAAVLHNIALA

DRNEDMEDYDNDIINEEERLIENAQNRHAGQRKRANMINNVFSNE
>CCG000718.1
MAACKDFEEFFPALCVALSEEVCHNESTADMVPELVGSAVVGLTDMSRIHRESEASFADEL
LAMDDKDFFQHLRLTKPAFTYLLDNIKKDDTPPRTHGKKKVSTTEGLLLTLWFLGNKTSFR
ETAFQFRRTISAVHRLFYSTVLLICELAKKVITWPSDIDMTQNEFVKIANIPGIVGAIDATHIN
VRPPEDNQKDYLDRMMNHSEVLLAVCDAKMRFTHISTGFPGSIHDQRCLDLSHNLSAAIK
TPPNEFFPRNELHLVGDSGFKLETALLVPYKDIGNLTEKQRMYNKKLSKSRVVIENAFGFLK
GRFRCLKHLEVDIENVTSIIVACCVIHNVALMFPDKLTVPELSEYCDDTDNEEHIFDPQSHA
VEKRNFICNNLP
>CCG038863.1
MAACKDFEEFFPALCVALSEEVCHNESTADMVPELVGSAVVGLTDMSRIHRESEASFADEL
LAMDDKDFFQHLRLTKPAFTYLLDNIKKDDTPPRTHGKKKVSTTEGLLLTLWFLGNKTSFR
ETAFQFRRTISAVHRLFYSTVLLICELAKKVITWPSDIDMTQNEFVKIANIPGIVGAIDATHIN
VRPPEDNQKDYLDRMMNHSEVLLAVCDAKMRFTHISTGFPGSIHDQRCLDLSHNLSAAIK
TPPNEFFPRNELHLVGDSGFKLETALLVPYKDIGNLTEKQRMYNKKLSKSRVVIENAFGFLK
GRFRCLKHLEVDIENVTSIIVACCVIHNVALMFPDKLTVPELSEYCDDTDNEEHIFDPQSHA
VEKRNFICNNLP
>CCG037687.1
MATGDHQMTLGDCHDVSQTTVSQCLKVVSRAIASLSRHYIQPPSGNDLQRTIQDFHAIHG
MPGVIGAIDCTHIAILRPSVENSEVFRCRKGFFSLNVQAVCGPDLRFHNVVARWPGSVHDS
RIFYNSRLCADIEENLNPRYHLLGDAGYALKRYLLTPVSVPTNEHERAYNNSHTHTRNTVE
RAFGVLKRRFGYLGKKVRTNLDTTKAIIVAAMVLHNIAVQTRLVLPQDGRDMINIDINAL
HNEVPIQRQANVLGRLKRQQIITDFF
>CCG039474.1
MSDDEFMDRFRVRKTSMYDLIEEIRDHLPAPNDSRGCPVPPHLQTLIAIRCMATGDHQMT
LGDCHDVSQTTVSQCLKVVSRAIASLSRHYIQPPSGNDLQRTIQDFHAIHGMPGVIGAIDCT
HIAILRPSVENSEVFRCRKGFFSLNVQAVCGPDLRFHNVVARWPGSVHDSRIFYNSRLCADI
EENLNPRYHLLGDAGYALKRYLLTPVSVPTNEHERAYNNSHTHTRNTVERAFGVLKRRFG
YLGKKVRTNLDTTKAIIVAAMVLHNIAVQTRLVLPQDGRDMINIDINALHNEVPIQRQAN
VLGRLKRQQIITDFF
>CCG080288.1
MATGDLHQTIGDCLDVSQQFVSSCSSHTVRAIAALYQRYVKFPGRHELGSIKTNFEAISGFP
GVIGAIDCTHIPILSPGGQQAETYRSRKGFFSLNVQAVCGPDLTFYNIICRWPGSVHDSRIFA
NSSLYAQLQAGDYNGHLLGDSAYPLRPFLVTPVGNPSRPNEGRYNLAHTKTRNVIERAFG
VWKRRFRCLAIPMRTSLDTTMATICSAAVLHNIAMADRNEDMEDYDNDIINEEERLIENA
QNRHAGQRKRET
>CCG026925.1
MATGDHQINLGDCHDVSQTVSQCLKVVSRAIASLSRHFIQPPSGSDLQRTIQDFHAIHGMP
GVIGAIDCTHIAILRPSVENSEVFRCRKGFFSLNVQAVCGPDLRFHNVVARWPGSVHDSRIF
YNSRLCADIEENLNPRYHLLGDAGHALKRYLLTPVSAPTNEHERAYNNSHTHTRNTVDR
AFGVLKRRFGYLGKKVRTNLDTTKAIIVAAMVLHNIAVQTRLVLPQDGRDMINIDINALH
NEVPIQRQANVLGRLKRQQIITDFF
>CCG037686.1
MPGVIGAIDCTHIAVLRPSVDNSEVFRCRKGFFSLNVQAVCGPDLRFHNVVARWPGSVHD

SRIFYNGRLCADIKENLNPRYHLLGDAGYALKRYLLTPVSVPTNEHERAYNNSHTHTRNT
VERAFGVLKRRFGYLGKKVRTNLDTTKAIIVAAMVLHNIAVQTRLVLPQDGRDMINIDIN
ALHNEVPIQRQANVLGRLKRQQIITDFF
>CCG021144.1
MSPNYIKFPEPANTRQVAEDFHAIASMPGVIGCIDGTLIPIVSPGGNTAELYRCRKGYFALN
VRAVCDAKMRFTNVISSWPGSVHDSRIFYNSRLCQKLEDEGYSGYLLGDSGYACKSYLLTP
ALDPQTEQENRYNASHIRTRNTIERCFGVWKRRFAVLRHLRTTLETSKKIIIAAAVLHNIAV
NSGMPALENVPEPCRSTGSCSSRRSNRPGAA
>CCG005913.1
MQHTVTLLAVCDARKKFTWIATCFAGSIHDQRCLSLSSMADKVASLPNQYFPQNSLHIVG
DSAFTLQAGLMVPYKDHGNLTQAQVIYNHNHSKTKCFIENTFGFLKNRFRCLAKLEVNFE
KAAPIIAACCILHNVALKFPDYLQEASTMDSPNASPSDSILVDSNANPNAAAKQRAICSML
S
>CCG057908.1
MAKAGRGQGFVRLHGRHDSTFSATLEEMDDLAFFQQLRLSKTAFNYLRSLLPQQEVVVHK
RGKKNTHTERVASTVCDARKKFTWIATGFAGSIHDQRCLSLSSMADKVASLPNQYFPQNS
LHIVGDSAFTLQAGLMVPYKDHGNLTQAQVIYNHKHSKTRCVIENTFGFLKNRFRCLAKL
EVNLEKAAPIIAACCILHNVALKFPDYLQEASTMDSPNASPSDSIQVDSNVNPNAAAKQR
ALCSMLS
>CCG064825.1
MLLDSDCEFGESDSSGDEYEPTQDELAEVETPIIPLVTRAATHGDTSGFVELPSSRRATEEAIK
MFNPWQQWEEFAAGQRALAALGRNTLRRERRVVPDRMNPFTAMSDREFTERFRLKKESV
NDIILKIGDCLPQSTDRRGCRVPPHLQVLLALAAMASGSHQEVIGDYFDVSQTTVSQCLAR
VASAIAGLIHQYITFPTGNMLHKVIEDFRAIAGMPGVVGCIDCTHFQIIKPPRNDSEIFKCKK
GYFSLNIQAICGPDLLFYNIVSRWPGSVDDASIFENSRIHDDLQDGILPGHLLGDSGYSNRQ
YLLTPLLSPNGPHEERYNASHIQTRNTIDRAFGVLKRRFAYLGKSIRTNLETTKAIIVASAILH
NIAVKTRLNMEDDINAVNENLQNNDLNVIENFVETRYDSDEVLQGRLKREQIIREHF
>CCG012692.1
MESSVALEEESVAFRLMKELREEDPATLRQWILLDREQFEELLAQVTPLIEKQDTNMRQTV
TPAERLTLTLRYLASEESYRSLSCQFRISHNLISTIVPSVCKAIYEVLRPRYVSLPRTEEEWQKV
ASDFYTQWNFPMCIGALDGKRILIQKPINSESSFYDYKGHFSVIMMALVDADCKFLYVDVG
SCGRASDGGVWDRCTLRQAVESDILNIPKPQTIPFTGTECPYVFVGDNAFPLKKYLMKPYP
GRGGTEESVIYNYRLSCARRISENAFGIMTARFQVFKQPIRIAPKHAEDITLAAVALHNTLR
DIYSPTELLDREDVGNGRLQQGQMHQHANAAMEGLHAVGRGHTNDAKEVRDTLKDYF
NNEGQVPWQAQVALLH
>CCG032195.2
MPELTKQKWEDVAKGFQKYADFPNCIGAIDGKHIRLTQPTGTGSLYYFKSFFSTVLLAVCD
ANYSFICVDIGAYGKSSDASIFNNSIMYQKLVENKLDIPGPKPISTAETCFLHVIVGDEAFGI
MENVMRTYSGKHLVHKKKIFNYILSRARRYIECTFGILANKWRIFHRPINVNIDFAEEIIKAC
CMLHNYVRARDGYRYEDTLFQVPLVGLPDINAPRGAMSANTARDKYADYFINEGKLAW
QDGMV
>CCG046226.1
MADCLEMSASSVCKFVHRIAVILARMSPNYIKFPEPANTRQVAEDFHAVAGMPGVIGCID
GTLIPIVSPGGNTAEIYRYRKGYLALIVMAVCDAKMRFTNVVSSWPGTVHDSRIFYNSRFCQ

KLEGEGYSGYLLGDSGYACKFYLLTLALDPQTEQENRYNASHIRTRDTIEKCFGVLKRRFAV
LRHLRTTLETSKKIIIAVAVLHNIAVNSGMPALENVPEPVEEVQDYALPEEATSRALRRRIIE
QWF
>CCG036054.1
MDQSSVSRAIDKAVGDTNNKIISYDTSYPGSTHDSFIWRHCALKQRFLAGHFGDALLIGDS
GYPLEPFLMTPVVHPTTPGEERYNQSHRRTRCIVERTFGILKSRFRCLHESGGSLQYDPEKT
MKIATSCMLLHNYCVDRRIPYVGDLVQEEEVPVQPVRDNRQVPGQVVRQEIIRNFFS
>New058565.1
MLITSFSVKYPGATNDAFIWRNCPLRDRFEAGTFRDFHLLGDCGYPLEPYLLTPFHDPMTE
GERQYNRSHKTTRVIIEQTFGVLKSRFRCLHRSGGSLQYDPKKCAKIAATCMWLHNRCIRR
RIPMIAPVGNDDGMNNDNDIIHGDHNPTGQDVRREIVEGFFT
>CCG052503.1
MAARALLAFLLAEQEDADDRARAALRRLRRSTKTSSRGVRCAGDYLRKGGMAAFYNLSD
FHTHFGMTRTQVEDLLNQLQPHYPYERGSKLPLENVVLASLWVLSSQESYRVIAERFQTGK
SVICASLHTFCNLVSGNLENHIRWPVVTAVKGTVRGFEELGFPGTLGAMGAFHITISKPKD
VEEPNEYMKETGVYYTTLLAVCNNKYKFTYVNVGHPGAFDNAEVFRRCELYKAFQEDPH
SLLPNNFHIGNKSYSYHILADGRFPLSEYVMTPYKDDHHLNAKEQEYNKRQLSAVCSISKAI
GVLKARFTRLKSLPMQHLAQCSVAIKACCILHNICVHTSDVDIYGANDMTNEMTTCTPIGF
ESNVTGENKRKAIADSFS
>CCG030379.1
MLITSFSVKYPGATNDAFIWRNCPLRDRFEAGTFRDFHLLGDSGYPLEPYLLTPFHDPMTEG
ERQYNRSHKITRVIIEQTFGVLKSRFRCLHRSGGSLQYDPKKCAKIAATSMWLHNRCIRRRI
PVIAPVGNDDGMNNDNDIIHGDHNPTGQDVRREIVEGFFT
>CCG035931.1
MFNNQWNVWRQWRKFQEDRHRIALNRSRRIVPDRMNPFTAMSDTEFVTRFRLRKESVNA
IIQEIQVQLPDSADRRGCRVPPHLQVLLTMAAMASGSHQMEVADSYDVSQQLISIVLARVS
RALAGLSREYVKFPSPNALTTVIEDFHGIAGMPGVIGCIDCTHIRIARPPREDSEVFSFYNIVV
RWPGSVHDAQIFEDSRVCNDLRDGLLPGHLLGDSGYGCRSYLLTPLVALNSVHEERYNAS
HARTRNTIERAFGVLKLRFSYLRNVIRTDLETTKAVIVAAVVLHNIAVQTRIDMSDEIRDVN
VDLQDVQINGIAKIDDAEPADDEVLLGCLKRDRIIRTHF
>CCG025547.1
MMALVDADCKFLYVDVGSCGRASDGGVWDRCTLRQAVESDILNIPKPQTIPFTGTECPYV
FVGDNAFPLKKYLMKPYPGRGGTEESVIYNYRLSCARRISENAFGIMTARFQVFKQPIRIAP
KHAEDITLAAVALHNTLREKSKDIYSPTELLDREDVGNGRLQQGQMHQHANAAMEGLH
AVGRGHTNDAKEVRDTLKDYFNNEGQVPWQAQMALLH
>CCG036055.1
MYRLCVTPNNKIISYDASYPGSTHDSFIWRHCALKQRFLAGHFGDALLIGDSGYPLEPFLMT
PVVHPTTPGEERYNQSHRRTRCIVERTFGILKVQCLQSQEEVCSTTPRRQ
>CCG052815.1
MQDNTLNIPDARPVSPNGDPLPFVLVGDEAFPLSTHMMRPYGGKYLSEMRKTFNYRISRA
RRYIECTFGILTNKWRILYRPIDVSVESAETFIRACCILHNFIRERDGVDFDNTLNVVGLDEG
DAVLQGNHRSALTIREKFAQ
>CCG014884.1
MMAALQVFEELDVLEDAAGERVRLPRRIVKDRLDLFLTLTEEEFTSRFRISKQSARALLADL

HLPEAADARGCPVPPHLQLLITLRWMATGDLHQTIGDCLDVSQQFLQAGDYNGHLLGDS
AYPLRPFLMTPVGNPSRPNEGRYNLAHAKTRNVIERAFGVWKRRFRCLAIPMRTSLDTTM
ATICSAAVLHNIAMADRNEDMEDYDNDIINEEERLIENAQNRHAGQRKRANMINNVFFK
>CCG071364.1
MAEAVTQAAAAVVFKNWSATMHVVANILYLSESSRPCASPQYPPRRASRQQQGGWTPH
QGAGKQAAVRVTGDAEMMAALQVFEELDVLEDAAGERVRLPRRIVKDRLDLFLTLTEEEF
TSRFRISKQSARALLADLHLPEAADARGCPVPPHLQLLITLRWMATGDLHQTIGDCLDVSQ
QFLQAGDYNGHLLGDSAYPLRPFLMTPVGNPSRPNEGRYNLAHAKTRNVIERAFGVWKR
RFRCLAIPMRTSLDTTMATICSAAVLHNIAMADRNEDMEDYDNDIINEEERLIENAQNRH
AGQRKRET
>CCG040943.1
MQQCSADDFGVSRATVSRIITQTVDALVTQENMRRFMGYLLNRGEQQRMKAEFAEIAGFP
GVVGAVDGTHVRIVAPHEHEEVYVNRKNYHSINVQVLFDAQYKLRAMVARWPGSTHDS
LNVCLHRGAVEYADGKKICAHIPYNEDTIAVFQLNINCLYL
>CCG022586.1
MAACKDFEEFFPALCVALSEEVCHNESTADMVPELVGSAVVGLTDMSRIHRESEASFADEL
LAMDDKDFFQHLRLTKPAFTYLLDNIKKDDTPPRTHGKKKVSTTEGLLLTLWFLGNKTSFR
ETAFQFRRTISAVHRLFYSTVLLICELAKKVITWPSDIDMTQNEFVKIANIPGIVGAIDATHIN
VRPPEDNQKDYLDRMMNHSEVLLAVCDAKMRFTHISTGFPGSIHDQRCLDLSHNLSAAIK
TPPNEFFSQE
>CCG000102.1
MKAEFAEIAGFPGMVGAVDGTHVRIVAPHEHEEVYVNRKNYHSINVQVLFDAQYKLWA
MVARWPGFTHDSLNVCLHRSVTEDADGTKIRAHFPYNEDTIAIFQLNINCLYL
>CCG082874.1
MTPVGNPSRPNEGRYNLAHAKTRNVIERAFGVWKRRFRCLAIPMRTSLDTTMATICSAAV
LHNIAWQTGMKIWKIMTMTL
>CCG065678.1
MPDQYHLKETSTLFLVGDEAFPLSTHMMRPYGGKYLLEMRKTFNYRISRARRPIDISVESAE
TFIRACCILHNFIRERDGVDFANTLNVVGLDEGDAVLQQGNHRSALTIREKFAQYFSSEEGS
VPWQA
>CCG026905.1
MTPVGNPSRPNEGRYNLAHAKTRNVIETAFGVWNRRLRCLAIPMRTSLDTTMATICSAAV
LHNIALADRDEDMEDYDNDIINEEEGLNENAQ
>Spa_10012569
MDLEEVACAWILHRRLKRRKRRERRYWVHPILQDRLTHGMYTTLYPSLREHEDKFFNYFR
MSVKSFDELLGLIQEEISSTNTLMRDAICPEEKLVITVRYLATGCSIADLHYGYRVGKSTISRIL
RQVCAAIWDRLKTMCMPEMTKQKWEDVAKGFQKYADFPNCIGAIDGKHIRLIQPTGTGS
LYYNYKSFFSTVLLAVCDANYSFLCVDIGAYGKSSDSAVFNNSILYQKLVENKLDIPGPKPIS
TAETTCFPHVIVGDEAFGIMENVMRPYSGRHLAHKKKIFNYRLSRARRYIECTFGILATKWR
IFHRPINVNIDFAEEIIKACCILHNYVRARDGYRYEDTLFQAPLVGLHDRNAPRGGMSANT
ARDKYADYFINEGKLAWQDGMV
>Spa_10015008
MDVEEVACVWLLHRRLKRRKQRQRQHWIHPILHDRLTHGLFTTLYPTLREHEPKFFNYCR
MSVKSFDDLLELIKEDISSTNTMMRDSICPEEKLVITLRYLATGCSIADLHYGYRLGKSTLAGI

LRQVCEAIWARMKTMCMPEMTKEMWEEVAKGFEKYAKFPNCIGAIDGKHIRLVQPKGSG
SLYYNYKLFFSTVLLAVCDANFSFIYVDIAAYGKSSDSAIFTESLLYKKLVENTLDVPEPKPISS
VETVCYPHVIVGDEAFGIMENVMRPYSGRHLTYRKKIFNYRLSRARRYIECTFGIMANKWRI
LHRPLNVNIDFAENIIKACCILHNYVRAREGYRYEDTLYRAPLVGLREGNVPRGGGSATST
RDRYADYFVNEGKLEWQDRMI
>Spa_10015495
MASRAVEKVFIAFFLAEEEAANNRAQVALRHLKQFKKENVRYLKTSRPQQVDTTAHMDE
HKAAVMREPKAAKRRRKGGRCAEDYLQKGGMMAFYNLSDFNTHFGMTRTQVEGLLNE
LQPHYPYERGSKLPLENVVLASLRVLSSQESYGMIAERFQTSKSVICTSLHTFCSLVSGNLEN
HIRWPVGNAIRGTIQGFEEVGFPGTLGAMDAFHIPINKPKDVQEPDEYMKENTMYCTTLL
AVCDNKYKFTYVNVGHPGAFDNSDVFKRCELHKAFQEDPDSLLPCDFHIGGKVYPFHIIA
DAGFPLSEYVMTPYADDGHLKHKEREYNRQHSSALLTVSKAVGVLKARYNRLKLLQMQH
LAQCSIAIKACCILHNICVHSSDAETYGVDEVPPLITAPCVHLESNVAGESKRSIIADSLFLE
>Spa_10012350
MYRLSKESFRELLSIIQGDLDRLDNRGRPLPAPYQLLLALHFYCTGSYQKEVGEQHGLEVSQ
PTVCRTIHRVSEALARRYGQFVVFPSTTQAPEIHERFYEVAQFPNVIGAIDCIHMRISNPGGT
MAEQCRNSKGWYSINCQVVVGPNLCILTAIVRWGGSVPDWLIYDNSRLRRVLEQGEYGHL
VGDAAYQCQRYLLTPVPCPSTHAERSYNRAHSSTRRRARQAFGMIRHRFQCTARELRSNP
KTSCAVILSCFALHNFVLQRQGPQEDAEAFYALDDVPQDRFSGECDPEGESYRQHIIHEWF
SSDAPEEVYQEERLGESRE
>Spa_10002192
MPIPAFYKLLKKTEPYTCKQDTSFRDSISPGARLEATLRFLAAGGSYTSLQYSTRISKQSLGLII
PETCEAIYNVLREDYLKGSHSIVLMAVVNANYEFMYVLVDVGANGRISDGGVWANSTLCT
RLQSGAMGLPPDEQLSDRHRTPPYVFVGDDAFPLKRYFMKPYPFKHRNNEQRIFSYRLSRA
RRVVENAFGIMSNKFRILLSAINLSPQKVEKIVFACTGLHNFLRREHESFYTPVGSLDNENV
VDGVVTPGAWRDDRQLLPLERLLRHPKNEAKAVRNEFMEYFNEEGAVPWQQRMCGIC
>Spa_10017339
MPFTAFHELLKKIDPYICKQDTGIRDSIPPGARLEATLRFLAAGGSYTSLQYSTRISKQSLGLM
IPETCAAIYNVLREDYLKGSHSIVLMAAVNANYEFLYVDVGTNGRVSDGGVWANTSLCAR
LQAGTAGLPADEQLSDSHRILPYVFLGDDAFPLKRYFMKPYPLKHQNNEQRVFSYRLSRAR
RVVENAFGIMANRFRVLLSTINLSPQKVEQIVLACTALHNFLRREHVSSYTRTPVDSFDNEN
IDDGVVTPGAWRDNRQLLPLDRIQRHPTNEAKDIRKEFMEYCNEEGAVPWQQRMCGIY
>Spa_10001345
MWHEVSPIWARGVARRDTRQEAQVLMAGILMVAAALLLQRRRLQQPPVPPQPRARRPRT
IWCREWLTRRSMHGDFHHLLQELNREDTKGYKNFLRIKPELFREMVDRLTPILAKKATRM
REPLSVGLKLAVTLQFLASGDSYTSLQYSFRVSKTAICRFVPKVCQAIIDIYKPEVLKCPRTPE
EWNQVAEGFSKRWNYHKCGGGLDGKHVRVKKPWHAGSLFFNYKKFHSIVLMAVADAN
YKFLYVDVGAEGSAGDGGTWFKCTLHDAIAQKRVGFPEHSFLPSDDTPIPFHVVADDAFA
LKTWLMKPYSHQSQVYEEKIFSYRLSRARRVVENAFGLLQSRFRVFGTTMLQRPAVVKIVT
MCGCVMHNLILDRYSNFHPQEVDCEDGDHNVIDGSWRNIPNLMQRLQTRRGTNPTREA
KAVRDYLALYYASEAGAVPWRERMVYPRGRPADEQRMEQ
>Spa_10004013
AGILPLLHLQHADEARLPRRLVKDHQNPFAAYSEEEFIRRYRLSKECIHTLLGQVEPDLPRA
KDGRGCSIPAYLQLLTAMMYFATGSFQICMADCLGMSAASVCKIVRSISTILCTLARTYIKFP

EPTDIPDLASNFFNIAGIPGCIGCIDGSNIICSWPGSVHDSRVFDNSRVCHQLEEGNYSGYLL
GDSGYPCRKYLLTPLLSPTTEKERKYNIAHIKTRNVIERAFGVLKHRFAILNKEVQTKLSTTN
IARLHNVPMPDEMDEDEVLGNAIPNINAEQAVPIENAASGLVLRSRIIENWF
>Spa_10000178
MMYFATGSFQICMANCLGMSAASVCKIVRSISTILCTLARTYIKFPEPTDIPDLASNFFNIAG
MPGCIGCIDGSYINIISPGGDHAELYRSRKGRFAINVMGICDYNLVGYLLGDSGYPCRKYLL
TPLLSPTTEKERKYNIAHIKTRNVIERAFGVLKRRFAILNKEVRTKLSTTKQIIMSCVILHNIA
RLHNVPMPDEMDEDEVLGNAIPNINAEQAVPIENAASGLVLRSHTIENWF
>Spa_10000148
MAEQQQQLPRRQRVYRQRRDVFNEYSDDELIKRFRMNRAGDSGYPCKEWLLTPYLNPLA
GVQTNYNTAHKRTRCVVERGIGQLKRRFHVLHGEVRLSPEKTCKIVYVCALLHNMCKQF
NIPVPINEEDEVFHDAAEGDVGADEEEPGEAEIPPNPGRPAGRNGHPFRDYIANLHFSKST
GHRDDLAPVKIRRLGSGKAMTQHPAALPLRYATIHNTKTTYVPLITIYRKHKA
>Spa_10013958
MAEQQQQLQRRQRVYRQQRDVFNKYNDDELIQRFRMNRAGDSGYPCKEWLLTPYLNPL
AGVQTNYNTAHKRTRCVVERGIGQLKRQFHVLHGEVRLSPEKTCKIVYVCALLHNMCKQ
FNIPVPINEEDELFHDAAEGDVGADEEEPGEAEIPSNPGRPAGRNGHPFRDYIANLHFRDA
VLCLNICDWCQSYFLNY
>Spa_10001228
MSAASVCKIVRSISTILCTLARTYIKFPEPTDIPDLASNFFNIAGMPGCIGCIDGSTSIKSPGGD
HEGNYSGYLLGDSGYPCRKYLLTPLLSPTTEKERKYNIAHIKTRNVIERAFGVLKRRFAILNK
EVRTKISTTKQIIMLCVILHNIARLHNVPMPDEMDEDEVLGNAIPNINAEQAVPIENVASGL
VLRSRIIENWF
>Spa_10004534
MWHEVSPIWVRGVARRDTRQEAQVLMAGILMVAAALLLRRRRLQQPPVPPQPRARRPRT
IWCREWLTRRSMHGDFHHLLQELNREDTKGYKNFLRIKPELFGEMVDRLTPILAKKATRM
REPLSGGLKLAVTLRFLASGDSYTSLQYSFRVSKTAICRFVPKVCQAIIDIYKPEVLKCPRTPE
EWNQVAEGFSKRWNYHKCGDANYKFLYVDVGAEGSAGDGGTWFKCTLHDAIAQKRVG
FPEHSFLPSDDTPIPFHIVADDAFALKTWLMKPYSHQSQVYEEKIFSYRLSRARRVVENAFG
LLQSRFRVFGTTMLQRPAVVKIVTMCGCVMHNLILDRYSNFHPQEVDCEDGDHNVIDGS
WRNIPNLMQRLQTRRGTNPAREAKAVRDYLALYYASEAGAVPWQERMVYPRGRPADEQ
RMEQ
>Spa_10009978
MYSLLGATHDAFVWSKCNLKQWFKRGEFGDFQLVGDSGYPFKPHLMVSLSNPTTDAEHR
YNRIHAQTRVIVEHTIGMLKSHFS
>Spa_10016699
MYSLLGATHDAFVWSNCNVKQRFEKGISFSLVSDSGYPLVPHLMVPLSNPTTDAEHRYNR
GHAQTRVIVEQTIWMLKSRFR
>Spa_10011597
MWHEVSPIWARGVARRDTRQEAQVLMAGILMVAAALLLRRRRLQQPPVPPQPRARRPRT
IWCREWLTRRSMHGDFHHLLQELNREDTKGYKNFLRIKPELFGEMVDRLTPILAKKATRM
REPLSVGLKLAVTLRFLASGDSYTSLQYSFRVSKTAICRFVPKVCEAIIDIYKPEVLKCPRTPEE
WNQVAEGFSKRWNYHKCGGGLDGKHVRVKKPWHAGSLFFNYKKFHSIVLMAVADANY
KFLYVDVGAEGSAGDGGTWFKCTLHDAIAQKRVGFPEHSFLPSDDTPIPFHIVADDAFALK

TWLMKPYSHQSQVYEEDGEHHRPTGHPHAIQGTDTQMYEYGALSWMSSAATNIQKLNA
VQRRALRLVAIDEEQQHPAPVTSLEHRRDVSALVVCHKTQVQRVPHLDPLRLLPHTVLRST
NLAPANTSAPTQPGLRGCGTCSRWPHPKCRTCPHTR
>MPC12324.1
MVAAQGNSMAALSRLQRLFALSELVELAELMGHQRPNKRRIVRHRLDPLQHHSNEEFLA
RYKLSKESFNALLEEITPHLPSSRDRRGLRISPSLQLLVTLRYLATGSFQLTVADTSEMSQASA
SRCIRRVVRAIAEVSAGHIRFPTPAEEGAVTQAFSAIAGMPECIGCIGGTLIPIKGPGGDDAE
LYRCRQGFFAINMTAVCDASLLVTNLVVNWPGSAHDSKIFNESRLRQTLEPGHYRGFLLG
HCGYPCHPYLLTPYADPKAPHEEKFNQAHARTHSCVERMFGILKRRFSILTTPLRTKRASRS
DIIVATVVLHNIAVRNGLPLEEGPEGRVEENVPVPDQENAAEGHIRRAEIAARFIS
>MPC26772.1
MMAFYNLSDFNTHFGMTRTQVEGLLNELQPHYPYERGSKLPLENVVLASLRVLSSQESYG
MIAERFQTSKSVICTSLHTFCSLVSGNLENHIRWPIGNAIRGTMQGFEDVGFPGTLGAMDAF
HIPINKPKDVQEPDEYMKENTMYCTTLLAVCDNKYKFTYVNVGHPGAFDDSDVFKRCEL
HKAFQEDPDSLLPYDFHIGGKVYPFHIIADAGFPLSEYVMTPYVDDGHLKHKEIEYNRHHS
SALLTVSKAVGVLKASAITTIYFIYVPLQHIDL
>MPC66609.1
MGPGDPQAEQFRCRKAIFSLNVQAVCGSDLNLYNIVCRWLGSVHDSRTFRNSAQYAQLQ
GGGYDGHLLRDSTYPFCPFLMTPIPIPRQQNEFRYNLAHAKTHNSIERAFGVLKRRFCCLSI
PMRTNMDKTTATVCSAAVLHNLGINFRDGYENLEDNDDGDDGCIENEYEDVIHDVQNR
QAGQRKRANIISSVF
>NP_001286523.1
MKRAATTKMTGATAAGATTTTSSTGAVGYPVLKTPKYVVQTSPSGSSGHQLQMLARKDT
QSLGVAINSLPPNTIIKATTRPSQTAPLTPNSAAVTPSTPSSSRNSTQSTPTVVPDARVSSAVR
QAVFIKRELPQPQRSMRNMTLGLVEQAPLLHLGVAPQHLSLLKRHICRNANVTHLDCCLT
LRKLKQNEHFALLAEHFELSESDVEDTFKRTLIKLARYLRPLIRWPDARHHNERFKHTPLN
YRANLLHVRSLIECVETDVPIDLGLGSGSYKFILCINTNGIISYVSSAFPGSCDDLQLFEASRFR
DVIPNYLTLCAEPGKAVRRARRSGFGDPHDSADEDEAAAEPKRSLTKFEAQRLSGQLASQ
QSLSVVDGALTSKRAPAIQLPTFNAQEPACRAQMRDMIDYLREFRMLDNSAIKQKSLLGYL
DEMIVVAAGLCNLKRQELES
>NP_611204.2
MALTITKFRTSRGMVVAVQPTVVLSPKIPDSQDNEKKPFSKSEPGNATVRVNAGGIVLNKIP
AIIRPSMKRAATTKMTGATAAGATTTTSSTGAVGYPVLKTPKYVVQTSPSGSSGHQLQMLA
RKDTQSLGVAINSLPPNTIIKATTRPSQTAPLTPNSAAVTPSTPSSSRNSTQSTPTVVPDARVS
SAVRQAVFIKRELPQPQRSMRNMTLGLVEQAPLLHLGVAPQHLSLLKRHICRNANVTHLD
CCLTLRKLKQNEHFALLAEHFELSESDVEDTFKRTLIKLARYLRPLIRWPDARHHNERFKH
TPLNYRANLLHVRSLIECVETDVPIDLGLGSGSYKFILCINTNGIISYVSSAFPGSCDDLQLFE
ASRFRDVIPNYLTLCAEPGKAVRRARRSGFGDPHDSADEDEAAAEPKRSLTKFEAQRLSGQ
LASQQSLSVVDGALTSKRAPAIQLPTFNAQEPACRAQMRDMIDYLREFRMLDNSAIKQKSL
LGYLDEMIVVAAGLCNLKRQELES
